# Association of Insurance Status with Health Outcomes Following Traumatic Injury: Statewide Multicenter Analysis

**DOI:** 10.5811/westjem.2015.1.23560

**Published:** 2015-03-17

**Authors:** Vatsal Chikani, Maureen Brophy, Anne Vossbrink, Khaleel Hussaini PhD, Chistopher Salvino, Jeffrey Skubic, Rogelio Martinez

**Affiliations:** * Arizona Department of Health Services, Bureau of Emergency Medical Services and Trauma System, Phoenix, Arizona; † West Valley Hospital, Goodyear, Arizona; ‡ Banner Good Samaritan Medical Center, Phoenix, Arizona

## Abstract

**Introduction:**

Recognizing disparities in definitive care for traumatic injuries created by insurance status may help reduce the higher risk of trauma-related mortality in this population. Our objective was to understand the relationship between patients’ insurance status and trauma outcomes.

**Methods:**

We collected data on all patients involved in traumatic injury from eight Level I and 15 Level IV trauma centers, and four non-designated hospitals through Arizona State Trauma Registry between January 1, 2008 and December 31, 2011. Of 109,497 records queried, we excluded 29,062 (26.5%) due to missing data on primary payer, sex, race, zip code of residence, injury severity score (ISS), and alcohol or drug use. Of the 80,435 cases analyzed, 13.3% were self-pay, 38.8% were Medicaid, 13% were Medicare, and 35% were private insurance. We evaluated the association between survival and insurance status (private insurance, Medicare, Medicaid, and self-pay) using multiple logistic regression analyses after adjusting for race/ethnicity (White, Black/African American, Hispanic, and American Indian/Alaska Native), age, gender, income, ISS and injury type (penetrating or blunt).

**Results:**

The self-pay group was more likely to suffer from penetrating trauma (18.2%) than the privately insured group (6.0%), p<0.0001. There were more non-White (53%) self-pay patients compared to the private insurance group (28.3%), p<0.0001. Additionally, the self-pay group had significantly higher mortality (4.3%) as compared to private insurance (1.9%), p<0.0001.

A simple logistic regression revealed higher mortality for self-pay patients (crude OR= 2.32, 95% CI [2.07–2.67]) as well as Medicare patients (crude OR= 2.35, 95% CI [2.54–3.24]) as compared to private insurance. After adjusting for confounding, a multiple logistic regression revealed that mortality was highest for self-pay patients as compared to private insurance (adjusted OR= 2.76, 95% CI [2.30–3.32]).

**Conclusion:**

These results demonstrate that after controlling for confounding variables, self-pay patients had a significantly higher risk of mortality following a traumatic injury as compared to any other insurance-type groups. Further research is warranted to understand this finding and possibly decrease the mortality rate in this population.

## INTRODUCTION

Multiple studies have shown insurance to be associated with health outcomes, including chronic diseases and medical complications.^1^,^2^ This has been extended to include outcomes of different traumatic injury subsets.^3^ Numerous investigations have examined the combined effect of race and insurance status on traumatic injury outcomes; however, some evidence suggests that insurance status alone may be a reliable predictor of mortality. While the general presupposition is that uninsured patients tend to be given the same level of intensive care services as insured patients, uninsured patients have exhibited higher odds of in-hospital mortality after both blunt and penetrating injuries as compared to insured patients with the same type of injury.^4^–^7^ Some evidence has shown similar associations between insurance status and mortality rates following traumatic injury among the pediatric population.^8^,^9^ Salim et al.^10^ found insured trauma patients tend to be older, female, more likely to have blunt traumatic injuries, and tend to have a higher injury severity when compared to uninsured patients.

There is some conflicting evidence as to whether insurance status is associated with mortality outcomes by injury type: blunt or penetrating. In one study where patients from a single hospital’s trauma registry were analyzed, Taghavi et al.^11^ found no difference in mortality between insured and uninsured patients with penetrating injuries. Conversely, in another study using National Trauma Data Bank (NTDB) data, when injury trauma type was restricted to blunt injury only, uninsured patients were found to have a significantly higher mortality compared to insured patients.^12^ Greene et al.^4^ found an association between insurance status and mortality rates, and hypothesized that the findings may be due to the fact that the uninsured patients were more likely to be involved in penetrating trauma; which is often a more lethal mechanism of injury.

The conflicting evidence persists when examining insurance status and different mechanisms of traumatic injury. Insurance status was not determined to be associated with mortality when a study by Rhee et al.^13^ restricted its sample solely to motor vehicle-related trauma patients. Clariadge et al.^14^ used data from a single hospital’s trauma registry where only penetrating injuries were analyzed, and reported no association with mortality when the cohort was limited to patients with spinal cord injuries. Perhaps lack of any significant association in these studies could be due to the fact that the study was limited to a single Level I trauma center and the results may have been due to their selective focus on a single regional facility. Schoenfeld et al.^15^ used national data and found both race/ethnicity and insurance status to be associated with higher mortality in spinal trauma patients.

According to U.S. Census Bureau^16^ statistics for 2006 through 2011, Arizona has consistently ranked above the national rate for uninsured adults under age 65. Statewide, 22.6 percent of all adults under age 65 have no health insurance coverage. Eight out of fifteen counties in Arizona have a higher percentage of adults under age 65 who do not have health insurance coverage compared to the state overall.^17^ Several studies that have examined the relationship between insurance status and trauma injury outcome have used data from either NTDB or a single hospital, neither of which is necessarily representative of the state/regional relationship between trauma injury and insurance status. Given the variation in access to care by region in Arizona the current study examines whether insurance status is associated with outcomes in blunt and penetrating trauma using state level trauma registry data. The inclusion of all ages, injury mechanisms, and trauma types in our study provides a more comprehensive picture of association between insurance status and mortality.

## METHODS

### Data and Sample

Our study involved a retrospective analysis of the Arizona State Trauma Registry (ASTR) data. Over the years, ASTR has received data from 23 designated trauma centers and four non-designated healthcare institutions - eight Level I trauma centers, and fifteen Level IV trauma centers. This manuscript was deemed exempt from human subjects review by the local board, as it is public health surveillance and does not publish any personally identifiable information.

The ASTR was queried to identify patients who had sustained blunt or penetrating trauma in 2008–2011. We excluded from the analysis cases with missing data on primary payer, sex, race, zip code of residence, injury severity score (ISS), and alcohol or drug use. Patients of Asian/Pacific Islander or “Other” race were excluded from the analysis due to their small sample size. Out of the 109,497 records queried, 80,435 (73.5%) met the inclusion criteria. The ASTR contains information on patient demographics, pre-hospital treatment, emergency department care, complications, ISS, hospital outcomes, charges, and complications.

### Measures

Overall mortality due to blunt and/or penetrating trauma was the primary outcome of interest. Other secondary outcomes included in-hospital mortality (i.e. excluded ‘dead on arrival’), total hospital length of stay (LOS), intensive care unit (ICU) LOS, discharge to rehabilitation centers (Skilled Nursing Facility, Long Term Care Facility, or Other Rehabilitation Facility), and mortality by mechanism of injury. The independent variable of interest in this study was payer status. We categorized patients based on their insurance status as follows: self-pay (patient designated as self-pay), Arizona Health Care Cost Containment System (AHCCCS i.e. State Medicaid), Medicare, and Private (includes Blue Cross/Blue Shield, no fault auto insurance, worker’s compensation, or other commercial plan). We classified external cause of injury codes (E-codes) into mutually exclusive categories of causes and intents of injury in accordance with the Centers for Disease Control and Prevention (CDC).^18^ Based on our sample size, mechanism of injury was classified into five categories, as opposed to using all 18 CDC recommended categories. These included (1) cut-pierce (injuries resulting from an incision, slash, perforation, or puncture by a pointed or sharp instrument, weapon, or object.); (2) falls; (3) firearm; (4) motor vehicle trauma (MVT); and (5) all other mechanisms. Intents of injury included the following four categories: unintentional, self-inflicted, assault, and undetermined/other.

Patient demographic variables included age, sex, race, ethnicity and median household income. We derived median household income data from the patient’s zip code of residence using 2011 Nielsen Claritas dataset that uses American Community Survey small area estimates. We also included known confounders and predictors for injury-related mortality, such as ISS, trauma type (blunt or penetrating) and drug and/or alcohol use (defined as any indication of use, including self-report, suspected use, or tested positive in hospital). ISS was categorized into four groups due to its nonlinear relationship with mortality: low (1–8), moderate (9–15), somewhat severe (16–24), and severe (25+).

### Analytic Procedures

We used bivariate and multivariate methods to compare risks for mortality at α =0.05. Mantel-Haenszel Chi-Square tests and logistic regression analyses were conducted using SAS v9.2 (SAS Institute, Inc., Cary, NC). We used ANOVA with Bonferroni correction to compare continuous variables across groups. Logistic regression analyses with adjusted odds ratios (OR) and 95% confidence intervals (95% CIs) were calculated for each of the independent variables.

## RESULTS

Insured patients accounted for 86.7% of the study population: 31,177 (38.8%) were Medicaid, 28,143 (35.0%) were private insurance and 10,418 (13.0%) were Medicare. The uninsured self-pay patients accounted for 13.3% (n=10,697) of the population. Most of the patients (89.0%) had blunt trauma. The population was predominantly male (65.2%), with a mean age of 36.6 years (standard deviation=22.6 years). Most of the patients were White (57.6%), followed by Hispanics (29.4%), American Indian/Alaska Native (8.3%), and Black/African American (4.7%).

Table 1 illustrates the general characteristics of the population by payer. The average patient age was 30.4 years for self-pay, 27.8 years for Medicaid, 72.0 years for Medicare, and 35.6 years for those with private insurance. Self-pay patients were more likely to be males (75.7%), Hispanic (40.3%), and less severely injured (ISS 0–8, 70.6%) as compared to the other insurance groups. More self-pay patients suffered from penetrating trauma (18.2%), and used drugs and/or alcohol (42.3%) as compared to other insurance groups.

Table 2 provides differences in survival status, discharge to rehabilitation, and LOS by payer. There was a significant difference in overall mortality and in-hospital mortality among the four groups, with Medicare patients having the highest mortality, followed by self-pay patients. The rate of discharge to rehabilitation also differed significantly among the four groups, with self-pay patients having the lowest rate of being discharged to a rehabilitation facility (1.49%). Self-pay patients had a significantly shorter overall LOS in the hospital after admission (median 1 day, IQR 0–2) as compared to the patients with private insurance (median 1 day, IQR 0–3, p<0.0001). Further, following a traumatic injury, self-pay patients remained in the intensive care unit (ICU) for a significantly shorter length of time (median 1 day, IQR 1–3) as compared to the patients with private insurance (median 2 days, IQR 1–4, p<0.0001).

Table 3 provides unadjusted and adjusted odds ratios for insurance status as associated with overall mortality, in-hospital mortality and rehabilitation rates. In the unadjusted model, both self-pay (OR=2.3, 95% CI [2.1–2.7]) and Medicare patients (OR=2.9, 95% CI [2.5–3.2]) had significantly higher odds of overall mortality as compared to patients with private insurance. It is evident that, even after adjusting for known predictors as well as demographic confounders (age, gender, race/ethnicity, ISS, trauma type, drug/alcohol use, and income), insurance status was still significantly associated with trauma related mortality. In the adjusted model, self-pay patients were approximately three times (i.e. OR=2.76, 95% CI [2.3–3.32]) more likely to die in a trauma-related incident compared to privately insured patients. Medicaid (OR=1.26, 95% CI [1.08–1.47]) as well as Medicare patients (OR=1.41, 95% CI [1.17–1.71]) also had higher mortality compared to privately insured patients. Appendix A compares multiple models, which add the covariates in a stepwise manner so as to assess the effect of these variables on relationship between mortality and insurance status. The first model is unadjusted, assessing only at insurance status and mortality. Model II removes insurance status and is an unadjusted assessment of the demographic covariates. Model III adjusts for overall LOS, ISS, trauma type, and substance use without adjusting for demographic covariates. Model IV (full adjusted model) keeps the previous covariates in the model, and adds age, sex, race/ethnicity, and median household income.

After excluding death on arrival to the emergency department (ED) from the analysis, similar results were found for in-hospital mortality. The self-pay (adjusted OR=2.16, 95% CI [1.74–2.67]), Medicare (adjusted OR=1.57, 95% CI [1.28–1.93]), and Medicaid (adjusted OR=1.26, 95% CI [1.06–1.49]) patients had significantly higher in-hospital mortality as compared to the privately insured patients. Of those patients who survived to discharge, self-pay patients were least likely to be discharged to a rehabilitation facility as compared to other insurance groups. The adjusted model revealed that self-pay patients had the significantly lower odds of being discharged to a rehabilitation facility than privately insured patients (OR=0.16, 95% CI [0.13–0.19]).

We further analyzed the adjusted model based on mechanism (Table 4) and intent of injury (Table 5) sub-groups. Self-pay patients had significantly higher fall-related mortality (OR=2.06, 95% CI [1.17–3.61]), firearm-related mortality (OR=2.72, 95% CI [1.59–4.64]), MVT-related mortality (OR=3.11, 95% CI [2.34–4.14]) and mortality related to all other mechanisms of injury (OR=2.59, 95% CI [1.62–4.15]), with the exception of cut-pierce. Further, self-pay status was significantly associated with mortality related to unintentional injuries (OR=3.19, 95% CI [2.57–3.96]) and mortality related to assaults (OR=2.76, 95% CI [2.3–3.32]).

## DISCUSSION

The higher odds of trauma-related mortality for self-pay patients may be related to a variety of factors. One possible explanation is care coordination in the trauma system, which is exacerbated by shorter LOS for this group. We know that LOS is proportional to costs and for a successful definitive care plan, it is important that the patient remains in the care of the trauma team to prevent further deterioration post-trauma and optimize conditions for recovery. However, in the case of self-pay patients, perhaps the high costs associated with post-injury care are prohibitive, thereby reducing the LOS and increasing the risk for mortality. A potential confounder for the increased odds of mortality may be due to pre-existing comorbidities in this group.^19^,^20^ Another factor that is perhaps attributable is potential differences in management of care (i.e. less use of procedural interventions).^19^ Interestingly, while alcohol and/or drug use have been reported to be risk factors for increased in-hospital complications and in-hospital mortality,^20^,^21^ we consistently found these to have a protective effect. Perhaps residual confounding and interaction with mechanism of injury may explain this effect; however, assessing these effects are beyond the scope of this paper.

Another finding of the analysis was an increased mortality in Medicare patients, which may be attributable to advanced age and underlying comorbid factors. However, controlling for these potential comorbid conditions is again beyond the scope of this paper, due to the lack of robust documentation in this field of the registry.

This topic will change dramatically with the implementation of the Affordable Care Act. However, it will take a few years to see the full effects on the healthcare system, and it will be interesting to see the effects of the variety of coverage options that are available under the new law on trauma-related mortality.

## LIMITATIONS

Despite the strong evidence of our findings, the study is limited in that the data are cross-sectional and no measures to account for pre-existing comorbidities were available. Additionally, a quarter of the study population was excluded due to missing values within the variables of interest. The state trauma system was still in the process of growing at the time this study was performed, and further research on this subject could be beneficial once the designation of new trauma centers slows down. Future research studies can examine the extent to which payer status has effect modification on LOS, injury severity, drug and alcohol, as well as race and/or ethnicity to explain trauma-related mortality. Our findings nonetheless draw attention to disparities that exist in definitive care for traumatic injuries among self-pay patients as compared to other insurance groups.

## CONCLUSION

The results of this study indicate that insurance status is associated with trauma-related mortality for the majority of the mechanisms and intents of injuries studied. The odds of mortality for self-pay patients were twice that of patients with private insurance. Our study findings add to existing literature on trauma-related mortality and payer status by using a statewide trauma registry database, and are consistent with other studies that found that uninsured patients had elevated rates of mortality.^3^–^6^,^8^–^10^,^12^,^15^ This information may aid in the development of targeted interventions aimed at reducing the high risk of trauma-related mortality in uninsured patients.

## Figures and Tables

**Table 1:** Characteristics of the population in the Arizona State Trauma Registry during 2008–2011 by payer.

Variables	Self-pay (n=10,697)	AHCCCS (n=31,177)	Medicare (n=10,418)	Private (n=28,143)	χ^2^ (p-value)
Age of the patient (+SD) in years	30.4 (14.5)	27.8 (17.3)	72 (14.9)	35.6 (20.1)	p<0.001
Male (%)	8,100 (75.7)	21,042 (67.5)	5,367 (51.5)	17,921 (63.7)	p<0.001
Non-Hispanic White (%)	5,029 (47.0)	12,676 (40.7)	8,428 (81.0)	20,175 (71.7)	-
Hispanic (%)	4,306 (40.3)	11,875 (38.1)	1,304 (12.5)	6,167 (21.9)	p<0.001
American Indian/Alaskan Native (%)	687 (6.4)	4,598 (14.8)	417 (4.0)	962 (3.4)	-
African American/Black (%)	675 (6.3)	2,028 (6.5)	269 (2.6)	839 (3.0)	-
Income <=$34,000 (%)	2,912 (27.22)	10,521 (33.75)	2,039 (19.57)	3,704 (13.16)	-
Income >$34,000 <= $45,000 (%)	3,185 (29.77)	10,084 (32.34)	3,351 (32.17)	7,752 (27.55)	p<0.001
Income >$45,000 <= $55,000 (%)	2,483 (23.21)	6,275 (20.13)	2,643 (25.37)	6,593 (23.43)	-
Income >$55,000 (%)	2,117 (19.79)	4,297 (13.78)	2,385 (22.89)	10,094 (35.87)	-
Injury severity score (ISS) <=8 (%)	7,547 (70.6)	19,959 (64.0)	4,618 (44.3)	17,645 (62.7)	-
ISS 9–15 (%)	1,972 (18.4)	6,764 (21.7)	3,332 (32.0)	6,448 (22.9)	p<0.001
ISS 16–24 (%)	624 (5.8)	2,650 (8.5)	1,632 (15.7)	2,513 (8.9)	-
ISS 25–75 (%)	554 (5.2)	1,804 (5.8)	836 (8.0)	1,537 (5.5)	-
Penetrating trauma (%)	1,948 (18.2)	4,726 (15.2)	465 (4.5)	1,692 (6.0)	p<0.001
Drug and alcohol use (%)	4,598 (42.3)	11,947 (38.3)	1,557 (15.0)	5,632 (20.0)	p<0.001
Median total length of stay (IQR) in days	1.0 (0, 2)	1.0 (0, 3)	3.0 (1, 6)	1.0 (0, 3)	-

*AHCCCS*, Arizona Health Care Cost Containment System; *ISS,* injury severity score

**Table 2:** Survival status and length of stay in the Arizona State Trauma Registry during 2008–2011 by payer.

	Payer status			
	
Outcome variables	Self-pay	AHCCCS	Medicare	Private insurance
Survival status†				
Overall mortality***	456 (4.26)	635 (2.04)	537 (5.15)	524 (1.86)
In-hospital mortality***	250 (2.38)	478 (1.54)	468 (4.52)	379 (1.35)
Discharge to rehabilitation facility ***	153 (1.49)	2,231 (7.30)	3,459 (35.01)	2,351 (8.51)
Length of stay‡				
Total length of stay in days median (IQR)	1 (0,2)***	1 (0,3)***	3 (1,6)***	1 (0,3) (Reference)
Intensive care unit length of stay (IQR)	1 (1,3)***	2 (1,4)***	2 (1,5)***	2 (1,4) (Reference)

*AHCCCS*, Arizona Health Care Cost Containment System

*** p<0.0001

† Mantel-Haenszel Chi-square tests

‡ ANOVA with Bonferroni correction test p<0.0167

**Table 3:** Self-pay as associated with mortality in Arizona State Trauma Registry during 2008–2011.

	Unadjusted odds ratio (95% CI)	Adjusted odds ratio (95% CI)
Overall mortality rate		
Private (reference)	1.00	1.00
Medicaid	1.10 (0.98, 1.23)	1.26 (1.08, 1.47)
Medicare	2.90 (2.54, 3.24)	1.41 (1.17, 1.71)
Self-pay	2.35 (2.07, 2.67)	2.76 (2.30, 3.32)
In-hospital mortality rate (excluding death on arrival)		
Private (reference)	1.00	1.00
Medicaid	1.14 (1.00, 1.31)	1.26 (1.06, 1.49)
Medicare	3.45 (3.01, 3.96)	1.57 (1.28, 1.93)
Self-pay	1.80 (1.51, 2.09)	2.16 (1.74, 2.67)
Rehabilitation rate (excluding all deaths)		
Private (reference)	1.00	1.00
Medicaid	0.85 (0.80, 0.90)	0.92 (0.84, 0.99)
Medicare	5.79 (5.46, 6.14)	1.68 (1.54, 1.82)
Self-pay	0.16 (0.14, 0.19)	0.16 (0.13, 0.19)

**Table 4:** Self-pay as associated with mortality by mechanism of injury in Arizona State Trauma Registry during 2008–2011.

Payer status	Cut-pierce	Falls	Firearm	Motor vehicle trauma	All other mechanisms
Private insurance (reference)	1.0	1.0	1.0	1.0	1.0
Self-pay	1.65 (0.54–5.05)	2.06 (1.17–3.61)^*^	2.72 (1.59–4.64)***	3.11 (2.34–4.14)***	2.59 (1.62–4.15)***
AHCCCS	0.89 (0.32–2.48)	1.28 (0.87–1.86)	1.1 (0.67–1.81)	1.51 (1.19–1.91)***	1.29 (0.87–1.89)
Medicare	0.61 (0.11–3.46)	1.32 (0.96–1.83)	1.28 (0.53–3.11)	1.82 (1.34–2.49)***	3 (1.74–5.18)***

*AHCCCS*, Arizona Health Care Cost Containment System

*** p <0.001

Estimates are odd ratios with CI in parentheses and all models are adjusted for covariates included in full model unless otherwise noted.

**Table 5:** Self-pay as associated with mortality by intent of injury in Arizona State Trauma Registry during 2008–2011.

Payer status	Unintentional	Self-inflicted	Assault	Undetermined/other
Private insurance (reference)	1.0	1.0	1.0	1.0
Self-pay	3.19 (2.57–3.96)***	0.63 (0.24–1.66)	2.07 (1.22–3.5)**	1.98 (0.75–5.22)
AHCCCS	1.39 (1.16–1.66)***	0.4 (0.18–0.89)*	0.9 (0.55–1.48)	1.55 (0.72–3.35)
Medicare	1.46 (1.19–1.79)***	0.63 (0.19–2.07)	2.21 (1.01–4.88)*	0.54 (0.22–1.29)

*AHCCCS*, Arizona Health Care Cost Containment System

*** p<0.001

** p<0.01

* p<0.05

Estimates are odd ratios with CI in parentheses and all models are adjusted for covariates included in full model unless otherwise noted.

## References

[1] Chen AY, Schrag NM, Halpern MT (2007). The impact of health insurance status on stage at diagnosis of oropharyngeal cancer. Cancer.

[2] Kelz RR, Gimotty PA, Polsky D (2004). Morbidity and mortality of colorectal carcinoma surgery differs by insurance status. Cancer.

[3] Haider AH, Chang DC, Efron DT (2008). Race and Insurance Status as Risk Factors for Trauma Mortality. Arch Surg.

[4] Greene WR, Oyetunji TA, Bowers U (2010). Insurance status is a potent predictor of outcomes in both blunt and penetrating trauma. Am J Surg.

[5] Tepas JJ, Pracht EE, Orban BL (2011). Insurance Status, Not Race, Is a Determinant of Outcomes for Vehicular injury. J Am Coll Surg.

[6] Haas JS, Goldman L (1994). Acutely Injured Patients with Trauma in Massachusetts: Differences in Care and Mortality, by Insurance Status. Am J Public Health.

[7] Marquez de la Plata C1, Hewlitt M, de Oliveira A (2007). Ethnic differences in rehabilitation placement and outcome after TBI. J Head Trauma Rehabil.

[8] Rosen H, Salah F, Lipsitz SR (2009). Lack of insurance negatively affects trauma mortality in US children. J Pediatr Surg.

[9] Hakmeh W, Barker J, Szpunar SM (2010). Effect of Race and Insurance on Outcome of Pediatric Trauma. Acad Emerg Med.

[10] Salim A, Ottochian M, DuBose J (2010). Does Insurance Status Matter at a Public Level I Trauma Center?. J Trauma.

[11] Taghavi S, Jayarajan SN, Duran JM (2012). Does payer status matter in predicting penetrating trauma outcomes?. Surgery.

[12] Weygant PL, Losonczy LI, Schneider EB (2012). Disparities in mortality after blunt injury: Does insurance type matter?. J Surg Res.

[13] Rhee PM, Grossman D, Rivara F (1997). The Effect of Payer Status on Utilization of Hospital Resources in Trauma Care. Arch Surg.

[14] Clariadge JA, Croce MA, Weinberg JA (2006). The real predictors of disposition in patients with spinal cord injuries. J Trauma.

[15] Schoenfeld AJ, Belmont PJ, See AA (2013). Patient demographics, insurance status, race and ethnicity as predictors of morbidity and mortality after spine trauma: a study using the National Trauma Data Bank. Spine J.

[16] U.S. Census Bureau Small Area Health Insurance Estimates: 2006–2011.

[17] Small Area Health Insurance Estimates Team (2012). US Census Bureau.

[18] Centers for Disease Control and Prevention External Cause-of-Injury (E-Code) Matrices: ICD-9.

[19] Mainous AG, Diaz VA, Everett CJ (2011). Impact of Insurance and Hospital Ownership on Hospital Length of Stay Among Patients With Ambulatory Care–Sensitive Conditions. Ann Fam Med.

[20] Rootman DB, Mustard R, Kalia V (2007). Increased incidence of complications in trauma patients cointoxicated with alcohol and other drugs. J Trauma.

[21] Chen CM, Yi HY, Yoon YH (2012). Alcohol Use at Time of Injury and Survival Following Traumatic Brain Injury: Results From the National Trauma Data Bank. J Stud Alcohol Drugs.

